# The Prevalence of Thyroid Abnormalities in Patients With Chronic Kidney Disease: A Cross-Sectional Study at a Tertiary Care Hospital

**DOI:** 10.7759/cureus.43065

**Published:** 2023-08-07

**Authors:** Rishav Raj, Vijay Kumar, Divendu Bhushan, Ratnadeep Biswas, Vishnu S Ojha

**Affiliations:** 1 Internal Medicine, All India Institute of Medical Sciences Patna, Patna, IND

**Keywords:** glomerular filtration rate, creatinine, urea, thyrotropin, triiodothyronine, thyroxine, hypothyroidism, thyroid diseases, renal insufficiency, chronic kidney disease

## Abstract

Background and objective

Chronic kidney disease (CKD) is a clinical syndrome characterized by the irreversible loss of kidney function. It is a widespread condition affecting populations worldwide. The kidneys play a crucial role in the metabolism, breakdown, and elimination of thyroid hormone and thyroid-stimulating hormone (TSH). Consequently, thyroid dysfunction can occur as an endocrine manifestation in CKD patients. Previous studies investigating thyroid abnormalities and the severity of CKD have yielded diverse outcomes. In light of this, this study aimed to determine the prevalence of thyroid dysfunction in CKD patients and explore the association between different thyroid dysfunctions and markers of kidney function.

Methods

A total of 140 CKD patients who met the inclusion criteria were recruited, and their demographic details and routine investigations were recorded. Blood samples were collected for kidney function tests and thyroid function tests. The primary outcome measures included markers of kidney function [urea, creatinine, and estimated glomerular filtration rate (e-GFR)] and thyroid profile [TSH, free thyroxine (FT4), and free triiodothyronine (FT3)]. Mean and standard deviation (SD) were calculated for continuous variables, while frequencies were calculated for categorical data. Fisher's exact test was employed to evaluate the association between two categorical variables, and p-values below 0.05 were considered statistically significant.

Results

The mean (± SD) urea, creatinine, and e-GFR were found to be 139 (± 81.1) mg/dL, 5.33 (± 4.1) mg/dL, and 20.1 (± 15) ml/min/1.73 m^2^, respectively. Of note, 133 (95%) patients had elevated urea levels, with the majority (n = 109, 77.8%) having urea levels between 40 and 199 mg/dL; 70 (50%) patients had creatinine levels less than 4 mg/dL, and 107 (76.4%) had e-GFR of less than 30 ml/min/1.73 m^2^. The mean (± SD) TSH, FT4, and FT3 levels were found to be 6.64 (± 11.2) mIU/ml, 13.6 (± 4.54) pmol/L, and 2.65 (± 1.89) pmol/L, respectively. It was observed that 18 (12.9%, 95% CI: 8.29-19.4%) of the CKD patients had hypothyroidism and 21 (15%, 95% CI: 10.02-21.8%) had subclinical hypothyroidism (SCH), while only two (1.4%, 95% CI: 0.39-5.05%) and five (3.6%, 95% CI: 1.5-8.08%) had hyperthyroidism and subclinical hyperthyroidism, respectively. Thirty-nine (27.9%, 95% CI: 21.1-35.8%) patients had low FT4 levels, whereas a considerable majority (n = 123, 87.9%, 95% CI: 81.41-92.28%) of the patients suffering from CKD were found to have low FT3 levels. The associations of urea levels with SCH, low FT4, and FT3 status were found to be statistically significant with p-values of 0.002, 0.033, and <0.001, respectively. The association between e-GFR and low FT3 status was also statistically significant, with a p-value of 0.014.

Conclusion

Nine out of 10 patients with CKD were discovered to have low FT3 levels, whereas one in four patients had low FT4 levels. The study participants also exhibited a significant presence of SCH and hypothyroidism, with prevalence rates of 15% and 12.9%, respectively. Urea levels and e-GFR, indicating the severity of CKD, showed a significant association with the presence of various thyroid abnormalities. Hypothyroidism in CKD patients can complicate disease progression, impact mortality rates, and affect overall quality of life. Therefore, routine screening for thyroid abnormalities should be conducted in all CKD patients.

## Introduction

Chronic kidney disease (CKD) is a clinical syndrome that occurs due to irreversible loss of kidney function, leading to metabolic, endocrine, excretory, and synthetic symptoms. This results in the accumulation of nitrogenous waste substances, causing metabolic abnormalities and distinct clinical manifestations. CKD is characterized by the presence of kidney damage or decreased glomerular filtration rate (GFR) (60 ml/min/1.73 m^2^ or less) for three or more months, irrespective of the cause [1]. It can result from diverse etiologies and pathological processes that damage the kidneys, such as diabetic nephropathy, glomerulonephritis, hypertension-associated renal disease (including vascular and ischemic kidney disease), autosomal dominant polycystic kidney disease, other cystic and tubulointerstitial kidney diseases, Alport disease, CKD of unknown etiology, etc. [2]. The etiologies may vary with the geographical region, race, and age of the patient.

CKD is a common disorder in most populations across the globe. The global burden of disease (GBD) study found that there were 698 million cases of CKD in 2017, with a 9% global prevalence rate among adults [3]. CKD was responsible for 7.3 million years lived with disability (YLDs), 28.5 million years of life lost (YLLs), and 35.8 million disability-adjusted life years (DALYs). China and India, with 132.3 and 115.1 million cases, respectively, represented one-third of the global burden of CKD in 2017 [3].

CKD can manifest with a wide range of signs and symptoms that affect multiple organ systems. These include renal symptoms such as nocturia, oliguria, edema, and hypertension. Endocrine and metabolic disturbances may lead to hyperkalemia, metabolic acidosis, hypocalcemia, hyperphosphatemia, hyperparathyroidism, and protein-energy malnutrition. Dermatological manifestations can include pruritus, skin pigmentation changes, uremic frost, and nephrogenic fibrosing dermopathy. Neurological symptoms may involve peripheral neuropathies, altered sense of smell and taste, sleep disturbances, muscle cramps, seizures, and asterixis. Cardiovascular complications encompass left ventricular hypertrophy, uremic cardiomyopathy, chronic heart failure, arrhythmias, and others. Hematological abnormalities can result in anemia of uremia, granulocyte and lymphocyte dysfunction, platelet dysfunction, bleeding, and coagulation abnormalities. Gastrointestinal symptoms may include hiccups, anorexia, nausea and vomiting, uremic fetor, and nutritional deficiencies [4,5].

The thyroid gland is another such entity that affects nearly all organ systems in the body, and the functions of the thyroid and kidney are closely interrelated. Thyroidal status affects kidney function right from the embryonic stage. Thyroid hormones influence general tissue growth as well as tubular functions, electrolyte handling, and neural input. Hyper- and hypo-functioning of the thyroid influence mature kidney function indirectly by affecting the cardiovascular system and the renal blood flow and directly by affecting glomerular filtration, electrolyte pumps, the secretory and absorptive capacity of the tubules, and the structure of the kidney [6]. The risk of nephropathy and cardiovascular events increases in type 2 diabetes mellitus with subclinical hypothyroidism (SCH) [7].

On the other hand, the kidney plays an important role in the metabolism, degradation, and excretion of thyroid hormone, thyroid-stimulating hormone (TSH), and thyrotropin-releasing hormone (TRH) [8,9]. Thyroid dysfunction is a commonly seen endocrine abnormality among CKD patients. It has been seen that in CKD, as the GFR falls, there is a higher chance of developing primary hypothyroidism and SCH [10]. Tumor necrosis factor α (TNFα) and interleukin-one (IL-1) are two inflammatory cytokines that prevent type one 5' deiodinase, an enzyme required for peripheral thyroxine (T4) to triiodothyronine (T3) conversion, from expressing itself in CKD patients. Reduced protein binding and metabolic acidosis in CKD may also contribute to low total T3 levels [11].

In general, hypothyroidism and chronic renal disease have a lot of clinical overlap. Both disorders, in addition to low total and plasma-free triiodothyronine (FT3) levels, show a variety of symptoms, such as cold intolerance, puffy appearance, dry skin, lethargy, fatigability, and constipation. Furthermore, end-stage kidney disease is associated with a significantly higher prevalence of goiter [12,13]. Despite these findings, the majority of uremic patients are still thought to be euthyroid, as shown by normal basal metabolic rate, free thyroxine (FT4), and TSH plasma concentrations, as well as normal tendon relaxation time, which leads to the underdiagnosis of thyroid dysfunction in CKD patients [14-16].

The prevalence of thyroid abnormalities in CKD has been estimated to range from 13% in early CKD to 70% in end-stage renal disease (ESRD), according to a few studies [17,18]. However, previous research has yielded conflicting results, leading to uncertainty in the diagnosis and treatment of many patients. In CKD patients, thyroid dysfunctions often go unnoticed, resulting in various comorbidities, as there is currently a lack of global data on the screening and prevalence of thyroid dysfunction in CKD patients.

Hence, this study was conducted with the aim of estimating the prevalence of thyroid dysfunction in patients with CKD and determining the association of various thyroid dysfunctions with markers of kidney function.

## Materials and methods

Study design and setting

This was an observational, analytical, cross-sectional study conducted in the Department of General Medicine, All India Institute of Medical Sciences, Patna, a tertiary care center in eastern India, after obtaining approval from the Institute Ethics Committee (IEC).

Study participants

The study involved patients who were diagnosed with CKD and were on conservative management. They were admitted to the department during the period of study. Written informed consent was obtained from all the participants.

Inclusion criteria

The inclusion criteria were as follows: individuals aged more than 18 years; patients who fulfilled the criteria for CKD and were on conservative management; patients with CKD provided they had an estimated glomerular filtration rate (e-GFR) of less than 60 ml/min/1.73 m^2^, which was calculated using the Chronic Kidney Disease Epidemiology Collaboration (CKD-EPI) equation [19].

Exclusion criteria

Patients who were undergoing peritoneal dialysis or hemodialysis were excluded from participation. Additionally, individuals with an active infection, determined based on clinical features and leukocytosis (white blood cell count greater than 11,000 per cubic millimeter), were not included. Patients with established inflammatory disorders, such as connective tissue disorders, myositis, or other rheumatological conditions, were also excluded. Women who were pregnant or lactating were not considered eligible for the study. Patients with known cases of malignant or hematological disorders, a history of anti-cancer drug intake, any known coagulation disorder, or chronic liver disease were also excluded. Furthermore, patients who refused to provide written informed consent were not included in the study. Other conditions, including recent surgery, trauma, burns, and the use of drugs that alter the thyroid profile (such as amiodarone, phenytoin, beta-blockers, dopamine, steroids, estrogen pills, and iodine-containing drugs), were also considered grounds for exclusion.

Sampling

The participants meeting the inclusion criteria were selected using a purposive sampling method.

Sample size

The prevalence of CKD in India shows wide variations according to geographical regions, and the reported prevalence varies from less than 1% to 13%. In this study, the prevalence was taken as 13%. With a confidence level of 95% and a margin of error of 5%, the minimum sample size was calculated as 174. Upon finite population correction, the final sample size was calculated as 140.

Study protocol

**Figure 1:**
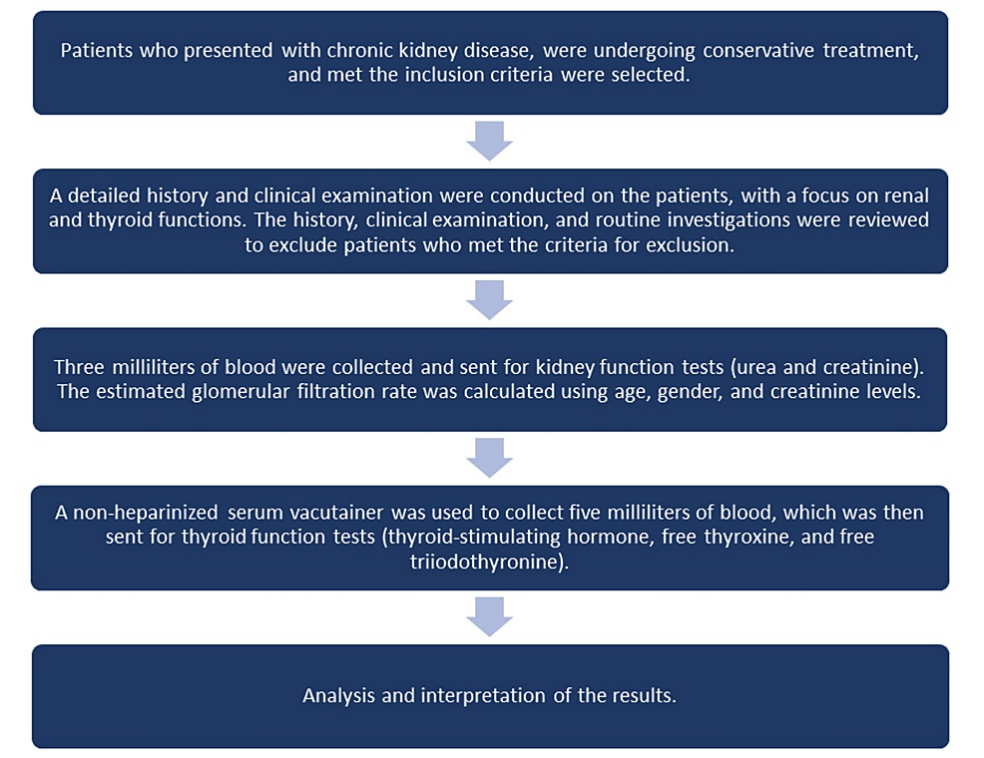
Flow diagram of the study protocol

Firstly, participants were selected based on a thorough assessment of their medical history, physical examination, and review of routine investigations. Following that, blood samples were collected from the participants for kidney and thyroid function tests. Subsequently, the obtained reports were analyzed (Figure 1).

Variables

The independent variables considered included age and sex. Markers of kidney function (urea, creatinine, and e-GFR) and thyroid profile (TSH, FT4, and FT3) were the primary outcome measures.

Definitions of thyroid abnormalities

The subjects were categorized as having overt hypothyroidism if they had a high serum TSH with a low FT4 concentration. Patients with high TSH with normal FT4 were classified as having SCH. Similarly, those with low TSH and high FT4 were considered to be cases of overt hyperthyroidism, while those with low TSH and normal FT4 were labeled as having subclinical hyperthyroidism.

Data collection

Structured questionnaires were administered to all the participants via direct interviews to record their demographic details. Reports were collected at the time of recruitment of the participants and after the generation of investigation reports, either directly from the patients or the hospital information system (HIS).

Statistical analysis

The analysis was performed using IBM SPSS Statistics, version 21.0 (IBM Corp., Armonk, NY). For the continuous variables, the minimum, maximum, mean, and standard deviation (SD) values were calculated. For the categorical variables, the frequencies were calculated. Fisher's exact test was employed to assess the association between two categorical variables, and p-values below 0.05 were regarded as statistically significant. Contingency tables and graphs were created where appropriate.

## Results

Of the total 140 participants in this study, 65 (46.4%) were 60 years of age or older, 63 (45%) were between the ages of 30 and 59 years, and only 12 (8.6%) were aged less than 30 years; 51 (36.4%) participants were females, while 89 (63.6%) were males (Table 1).

**Table 1:** Demographic details of the study participants (N = 140)

Variable	Categories	Frequency (n)	Percentage (%)
Age, years	<30	12	8.6
	30 – 59	63	45
	≥60	65	46.4
Gender	Male	89	63.6
	Female	51	36.4

A descriptive analysis was performed on the various lab parameters, and the mean values and SD were calculated for the continuous variables (Table 2).

**Table 2:** Descriptive analysis of various lab parameters (N = 140)

Serial number	Parameter	Unit	Reference range	Minimum	Maximum	Mean	Standard deviation
1	Urea	mg/dL	13 – 43	10.2	434	133	81.1
2	Creatinine	mg/dL	0.7 – 1.3	1.36	20.9	5.33	4.10
3	Estimated glomerular filtration rate	mL/min/1.73 m^2^	>90	2.09	59.2	20.1	15
4	Thyroid-stimulating hormone	mIU/L	0.35 – 5.49	0.01	83.5	6.64	11.2
5	Free thyroxine	pmol/L	11.5 – 22.7	0.44	26.6	13.6	4.54
6	Free triiodothyronine	pmol/L	3.5 – 6.5	0.22	22	2.65	1.89
7	Triglycerides	mg/dL	<150	36.6	636	178	100
8	Low-density cholesterol	mg/dL	100 – 129	6	326	84.2	54.2
9	High-density cholesterol	mg/dL	40 – 60	2.7	91.6	27.6	15.7
10	Total cholesterol	mg/dL	<200	34.3	426	148	70
11	Hemoglobin	g/dL	12 – 15	2.7	13.5	9.05	2.02
12	Glycated hemoglobin	%	4 – 5.6	3.7	14.6	6.56	1.95

When comparing the kidney function test reports among the study participants, the majority (n = 75, 53.6%) had urea levels <120 mg/dL, followed by 41 (29.3%) in the range of 120-199 mg/dL, and 24 (17.1%) had urea levels >200 mg/dL. The mean (± SD) urea among the study participants was 139 (± 81.1) mg/dL. Seventy (50%) subjects had creatinine levels <4 mg/dL, followed by 40 (28.6%) in the range of 4 to 7 mg/dL, 17 (12.1%) in the range of 8 to 12 mg/dL, and 13 (9.3%) had creatinine levels ≥12 mg/dL. The mean (± SD) creatinine level was 5.33 (± 4.1) mg/dL. Sixty-five (46.4%) individuals had e-GFR <15 ml/min/1.73 m^2^, while 42 (30%) had e-GFR between 15 and 29 ml/min/1.73 m^2^ and the remaining 33 (23.6%) had e-GFR in the range of 30 to 60 ml/min/1.73 m^2^. The mean e-GFR level was 20.1 (± 15) ml/min/1.73 m^2^.

Regarding thyroid profiles, it was observed that 94 (67.1%) individuals had TSH in the normal range, while 39 (27.9%) had TSH values ≥5.5 mIU/L, and only seven (5%) had TSH values <0.35 mIU/L. The mean (± SD) TSH level was 6.64 (± 11.2) mIU/L. Of note, 98 (70%) participants had FT4 values in the normal range, while 39 (27.9%) had FT4 values <11.5 pmol/L and the remaining three (2.1%) had FT4 values >22.7 pmol/L. The mean (± SD) FT4 level was 13.6 (± 4.54) pmol/L. Of note, 123 (87.9%) individuals had FT3 <3.5 pmol/L, while 16 (11.4%) had FT3 values in the normal range, and just one (0.7%) patient had FT3 >6.5 pmol/L. The mean (± SD) FT3 level was 2.65 (± 1.89) pmol/L (Table 3).

**Table 3:** Frequency distribution of markers of kidney and thyroid function (N = 140)

Parameter	Categories	Frequency (n)	Percentage (%)
Urea, mg/dL	<120	75	53.6
	120 – 199	41	29.3
	≥200	24	17.1
Creatinine, mg/dL	>4	70	50
	4 – 7	40	28.6
	8 – 12	17	12.1
	≥12	13	9.3
Estimated glomerular filtration rate, mL/min/1.73 m^2^	<15	65	46.4
	15 – 29	42	30
	30 – 60	33	23.6
Thyroid-stimulating hormone, mIU/L	<0.35	7	5
	0.35 – 5.4	94	67.1
	≥5.5	39	27.9
Free thyroxine, pmol/L	<11.5	39	27.9
	11.5 – 22.6	98	70
	≥22.7	3	2.1
Free triiodothyronine, pmol/L	<3.5	123	87.9
	3.5 – 6.4	16	11.4
	≥6.5	1	0.7

Among the 140 CKD patients, 18 (12.9%, 95% CI: 8.29 - 19.4%) had hypothyroidism and 21 (15%, 95% CI: 10.02 - 21.8%) had SCH, while only two (1.4%, 95% CI: 0.39 - 5.05%) subjects had hyperthyroidism and five (3.6%, 95% CI: 1.5 - 8.08%) had subclinical hyperthyroidism. Thirty-nine (27.9%, 95% CI: 21.1 - 35.8%) participants had low FT4 levels, while 123 (87.9%, 95% CI: 81.41 - 92.28%) had low FT3 levels (Table 4).

**Table 4:** Prevalence of thyroid abnormalities among the study participants (N = 140)

Thyroid abnormality	Frequency (n)	Percentage (%)	95% confidence interval
Hypothyroidism	18	12.9	8.29 – 19.4
Subclinical hypothyroidism	21	15	10.02 – 21.8
Hyperthyroidism	2	1.4	0.39 – 5.05
Subclinical hyperthyroidism	5	3.6	1.5 – 8.08
Low free thyroxine	39	27.9	21.1 – 35.8
Low free triiodothyronine	123	87.9	81.41 – 92.28

Fisher’s exact tests were carried out to look for associations of urea levels, creatinine levels, and e-GFR levels with hypothyroidism, SCH, low FT4, and low FT3 status. Due to the very low prevalence of hyperthyroidism and subclinical hyperthyroidism, they were not considered in these analyses.

About 20.8% of study participants with urea levels greater than 200 mg/dL had hypothyroidism, whereas among those who had urea levels between 120 and 199 mg/dL and those with less than 120 mg/dL, 7.3% and 13.3% had hypothyroidism, respectively. The association between urea and hypothyroidism status was not found to be statistically significant (p = 0.260). We found that 23.1% of participants with creatinine levels more than 12 mg/dL had hypothyroidism, while only 17.6%, 10%, and 11.4% had hypothyroidism among those who had creatinine between 8 and 11 mg/dL, 4 to 7 mg/dL, and less than 4 mg/dL, respectively. The association between creatinine and hypothyroidism status was not found to be statistically significant (p = 0.503). It was found 15.4% of patients with e-GFR less than 15 ml/min/1.73 m^2^ had hypothyroidism, and among those with e-GFR between 15 and 29 ml/min/1.73 m^2^ and 30 to 60 ml/min/1.73 m^2^, 11.9% and 9.1% had hypothyroidism, respectively. The association between the e-GFR and hypothyroidism status was not statistically significant (p = 0.725) (Table 5).

**Table 5:** Association between hypothyroidism and kidney function parameters (N = 140)

Variable	Categories	Hypothyroidism		P-value
		No, n (%)	Yes, n (%)	
Urea, mg/dL	<120	65 (86.7%)	10 (13.3%)	0.260
	120 – 199	38 (92.7%)	3 (7.3%)	
	≥200	19 (79.2%)	5 (20.8%)	
Creatinine, mg/dL	<4	62 (88.6%)	8 (11.4%)	0.503
	4 – 7	36 (90%)	4 (10%)	
	8 – 12	14 (82.4%)	3 (17.6%)	
	≥12	10 (76.9%)	3 (23.1%)	
Estimated glomerular filtration rate, mL/min/1.73 m^2^	<15	55 (84.6%)	10 (15.4%)	0.725
	15 – 29	37 (88.1%)	5 (11.9%)	
	30 – 60	30 (90.9%)	3 (9.1%)	

About 20.8% of study participants with urea levels greater than 200 mg/dL had SCH, whereas among those who had urea levels between 120 and 199 mg/dL and those with less than 120 mg/dL, 19.5% and 10.7% had SCH, respectively. The association between urea and SCH status was not found to be statistically significant (p = 0.308). We found that 23.1% of participants with creatinine levels more than 12 mg/dL had SCH, while only 23.5%, 15%, and 11.4% had SCH among those who had creatinine between 8 and 11 mg/dL, 4 to 7 mg/dL, and less than 4 mg/dL, respectively. The association between creatinine and SCH status was not found to be statistically significant (p = 0.437). It was observed that 15.2% of patients with e-GFR less than 15 ml/min/1.73 m^2^ had SCH, and among those with e-GFR between 15 and 29 ml/min/1.73 m^2^ and 30 to 60 ml/min/1.73 m^2^, 7.1% and 20% had SCH, respectively. The association between the e-GFR and SCH status was not statistically significant (p = 0.202) (Table 6).

**Table 6:** Association between subclinical hypothyroidism and kidney function parameters (N = 140)

Variable	Categories	Subclinical hypothyroidism		P-value
		No, n (%)	Yes, n (%)	
Urea, mg/dL	<120	67 (89.3%)	8 (10.7%)	0.308
	120 – 199	33 (80.5%)	8 (19.5%)	
	≥200	19 (79.2%)	5 (20.8%)	
Creatinine, mg/dL	>4	62 (88.6%)	8 (11.4%)	0.437
	4 – 7	34 (85%)	6 (15%)	
	8 – 12	13 (76.5%)	4 (23.5%)	
	≥12	10 (76.9%)	3 (23.1%)	
Estimated glomerular filtration rate, mL/min/1.73 m^2^	<15	52 (80%)	13 (20%)	0.202
	15 – 29	39 (92.9%)	3 (7.1%)	
	30 – 60	28 (84.8%)	5 (15.2%)	

About 50% of study participants with urea levels greater than 200 mg/dL had low FT4 levels, whereas among those who had urea levels between 120 and 199 mg/dL and those with less than 120 mg/dL, 24.4% and 22.7% had low FT4, respectively. The association between urea and low FT3 status was found to be statistically significant (p = 0.037). We observed that 46.2% of participants with creatinine levels more than 12 mg/dL had low FT4, while 35.3%, 32.5%, and 20% had low FT4 among those who had creatinine between 8 and 11 mg/dL, 4 to 7 mg/dL, and less than 4 mg/dL, respectively. The association between creatinine and low FT4 status was not found to be statistically significant (p = 0.141). It was seen that 36.9% of patients with e-GFR less than 15 ml/min/1.73 m^2^ had low FT4, and among those with e-GFR between 15 and 29 ml/min/1.73 m^2^ and 30 to 60 ml/min/1.73 m^2^, 21.4% and 18.2% had low FT4, respectively. The association between the e-GFR and low FT4 status was not statistically significant (p = 0.080) (Table 7).

**Table 7:** Association between low free thyroxine and kidney function parameters (N = 140) *Statistically significant

Variable	Categories	Low free thyroxine		P-value
		No, n (%)	Yes, n (%)	
Urea, mg/dL	<120	58 (77.3%)	17 (22.7%)	0.037*
	120 – 199	31 (75.6%)	10 (24.4%)	
	≥200	12 (50%)	12 (50%)	
Creatinine, mg/dL	>4	56 (80%)	14 (20%)	0.141
	4 – 7	27 (67.5%)	13 (32.5%)	
	8 – 12	11 (64.7%)	6 (35.3%)	
	≥12	7 (53.8%)	6 (46.2%)	
Estimated glomerular filtration rate, mL/min/1.73 m^2^	<15	41 (63.1%)	24 (36.9%)	0.080
	15 – 29	33 (78.6%)	9 (21.4%)	
	30 – 60	27 (81.8%)	6 (18.2%)	

About 95.8% of study participants with urea levels greater than 200 mg/dL had low FT3 levels, whereas among those who had urea levels between 120 and 199 mg/dL and those with less than 120 mg/dL, 97.6% and 80% had low FT3, respectively. The association between urea and low FT3 status was found to be statistically significant (p = 0.010). Of note, 92.3% of participants with creatinine levels more than 12 mg/dL had low FT3, while 100%, 92.5%, and 81.4% had low FT3 among those who had creatinine between 8 and 11 mg/dL, 4 to 7 mg/dL, and less than 4 mg/dL, respectively. The association between creatinine and low FT3 status was not found to be statistically significant (p = 0.124). We found that 95.4% of patients with e-GFR less than 15 ml/min/1.73 m^2^ had low FT3, and among those with e-GFR between 15 and 29 ml/min/1.73 m^2^ and 30 to 60 ml/min/1.73 m^2^, it was seen that 85.7% and 75.8% had low FT3, respectively. The association between the e-GFR and low FT3 status was statistically significant (p = 0.014)(Table 8).

**Table 8:** Association between low free triiodothyronine and kidney function parameters (N = 140) *Statistically significant

Variable	Categories	Low free triiodothyronine		P-value
		No, n (%)	Yes, n (%)	
Urea, mg/dL	<120	15 (20%)	60 (80%)	0.010*
	120 – 199	1 (2.4%)	40 (97.6%)	
	≥200	1 (4.2%)	23 (95.8%)	
Creatinine, mg/dL	<4	13 (18.6%)	57 (81.4%)	0.124
	4 – 7	3 (7.5%)	37 (92.5%)	
	8 – 12	0 (0%)	17 (100%)	
	≥12	1 (7.7%)	12 (92.3%)	
Estimated glomerular filtration rate, mL/min/1.73 m^2^	<15	3 (4.6%)	62 (95.4%)	0.014*
	15 – 29	6 (14.3%)	36 (85.7%)	
	30 – 60	8 (24.2%)	25 (75.8%)	

## Discussion

The current study aimed to determine the prevalence of thyroid dysfunction in CKD patients. A total of 140 patients with CKD who were on conservative management and fulfilled the inclusion criteria were recruited. Among them, 89 (63.6%) were male, and 128 (91.4%) participants were over the age of 30 years. The mean (± SD) urea, creatinine, and e-GFR were found to be 139 (± 81.1) mg/dL, 5.33 (± 4.1) mg/dL, and 20.1 (± 15) ml/min/1.73 m^2^, respectively. It was observed that 133 (95%) patients had elevated urea levels, with the majority (n = 75, 53.6%) having urea levels less than 120 mg/dL; 70 (50%) people had creatinine levels less than 4 mg/dL, and 107 (76.4%) had e-GFR less than 30 ml/min/1.73 m^2^.

The mean (± SD) TSH, FT4, and FT3 levels were found to be 6.64 (± 11.2) mIU/L, 13.6 (± 4.54) pmol/L, and 2.65 (± 1.89) pmol/L, respectively. It was observed that 18 (12.9%, 95% CI: 8.29-19.4%) of the CKD patients had hypothyroidism and 21 (15%, 95% CI: 10.02-21.8%) had SCH, while only two (1.4%, 95% CI: 0.39-5.05%) and five (3.6%, 95% CI: 1.5-8.08%) patients had hyperthyroidism and subclinical hyperthyroidism, respectively. We found that 39 (27.9%, 95% CI: 21.1-35.8%) patients had low free thyroxine levels, whereas a significant majority (n = 123, 87.9%, 95% CI: 81.41-92.28%) of the patients suffering from CKD were found to have low free triiodothyronine levels. The associations of urea levels with low FT4 and FT3 status were found to be statistically significant with p-values of 0.037 and 0.010, respectively. The association between e-GFR and low FT3 status was also statistically significant (p = 0.014). It was interesting to observe that despite the significant association of urea levels and e-GFR with low FT3 status, the relationship between creatinine and low FT3 status was not statistically significant, even though e-GFR is primarily calculated using creatinine. This lack of significance could be attributed to the relatively small sample size in our study. With a larger sample size, it is possible that the association could become statistically significant.

Numerous prior studies have investigated thyroid abnormalities and the severity of CKD, but the outcomes of these studies vary greatly. In our study, it was observed that hypothyroidism and SCH were much more common than hyperthyroidism or subclinical hyperthyroidism. This finding is in accordance with previous studies that report that hypothyroidism and SCH are much more common among pre-dialysis patients with CKD [20,21]. One study conducted by Kaptein et al. [12] revealed that iodide absorption by the thyroid gland is enhanced in CKD due to reduced iodide excretion. This leads to an elevation in plasma inorganic iodide levels, which can inhibit thyroid hormone production (known as the Wolff-Chaikoff effect). This phenomenon may explain the higher frequency of hypothyroidism and SCH observed. Similarly, the study by Alshammari et al. [22] reported that a considerable proportion of CKD patients had hypothyroidism. Since hypothyroidism is associated with a higher risk of mortality and adverse effects on health-related quality of life, these findings highlight the importance of recognizing and managing hypothyroidism in CKD patients [23].

We observed that the most common thyroid dysfunction was the presence of low FT3, affecting approximately nine out of every 10 patients with CKD. As it has been previously reported that the plasma reverse triiodothyronine (rT3) levels generally remain normal in such patients, it can be inferred that the low FT3 levels are not connected to an enhanced conversion of T4 to the metabolically inactive rT3 (often seen in euthyroid sick syndrome in which the type 2 and 3 deiodinases are upregulated) [24], but are most probably due to the diminished conversion of T4 to T3 in the periphery. This finding distinguishes uremic patients from individuals with chronic illnesses, as, unlike the former group, patients with chronic illnesses typically exhibit increased rT3 levels, which can serve as a protective mechanism for protein conservation [12,15,24].

Approximately one in every four patients was found to have low FT4 levels. Investigations conducted by Kaptein et al. [12], Singh et al. [25], Ramírez et al. [26], Scanlon et al. [27], and Witzke et al. [28] support the notion that uremia is associated with abnormalities in both the pituitary gland and the thyroid gland. These abnormalities may affect intrathyroidal processes and pituitary function. Furthermore, in CKD, there are alterations in the circadian rhythm of TSH and glycosylation of TSH, which can potentially impair the bioactivity of TSH. This could be the cause of the low FT4 levels observed, even though the majority of individuals in our study exhibited TSH levels within the normal range.

It has also been reported that patients with chronic kidney disease often exhibit enlarged thyroid glands [6]. The exact mechanism behind this enlargement remains unclear. It is also worth noting that there is a slightly increased prevalence of thyroid nodules and thyroid cancer in individuals with chronic renal impairment [12,29]. However, it seems unlikely that the minor alterations in TSH mentioned earlier will be sufficient to account for this transformation. Thus, it is possible that kidney failure might be linked to the accumulation of an unidentified goitrogenic substance [26].

The associations of urea levels with low FT4 and FT3 status were found to be statistically significant. The association between e-GFR and low FT3 levels was also found to be statistically significant, indicating that individuals with a low GFR tend to have lower FT3 levels. This finding aligns with the results of the study by Swaminathan et al. [30]. However, no significant association was observed between e-GFR and low FT4 levels. This contrasts with the findings of Ramírez et al. [26], who established a linear correlation between mean serum FT4 levels and the severity of CKD; in our study, the association was only found with FT3 and not FT4, suggesting the need for further evaluation of this relationship. It is worth noting that there is limited literature exploring thyroid abnormalities in CKD patients, emphasizing the need for additional research to determine their clinical significance and establish any statistical correlations among these variables.

This study has certain limitations. Firstly, thyroid dysfunction was examined in patients with CKD regardless of the specific etiology of CKD, thus preventing the investigation of individual correlations between CKD etiology and thyroid dysfunction. Additionally, thyroid dysfunction was not assessed in patients undergoing dialysis, as dialysis itself can independently influence thyroid profiles in individuals with CKD. Therefore, the findings of this study are not applicable to patients undergoing dialysis. Since it was a hospital-based study conducted at a single tertiary care hospital, the generalizability of the findings may be limited. Moreover, the sample size was determined based on the finite population, considering the prevailing circumstances related to the coronavirus disease 2019 (COVID-19) pandemic. Hence, further studies on this subject are necessary and should strive to address and mitigate these limitations.

## Conclusions

An astonishing nine out of 10 patients with CKD were discovered to have low triiodothyronine levels, whereas one in four patients displayed low thyroxine levels. The patients also exhibited a significant presence of SCH and hypothyroidism, with prevalence rates of 15% and 12.9%, respectively. Urea levels and e-GFR, indicating the severity of CKD, showed a significant association with the presence of various thyroid abnormalities. The presence of hypothyroidism in CKD patients can complicate disease progression, impact mortality rates, and affect overall quality of life. Hence, routine screening for thyroid abnormalities should be conducted in all CKD patients.
